# *N*′-[(*E*)-4-(Dimethyl­amino)­benz­ylidene]-2-(5-meth­oxy-2-methyl-1*H*-indol-3-yl)acetohydrazide

**DOI:** 10.1107/S1600536812026013

**Published:** 2012-06-13

**Authors:** Shaaban K. Mohamed, Peter N. Horton, Mehmet Akkurt, Mustafa R. Albayati, Antar A. Abdelhamid

**Affiliations:** a Chemistry and Environmental Division, Manchester Metropolitan University, Manchester, M1 5GD, England; b School of Chemistry, University of Southampton, Highfield, Southampton, SO17 1BJ, England; c Department of Physics, Faculty of Sciences, Erciyes University, 38039 Kayseri, Turkey; d Kirkuk University, College of Science, Department of Chemistry, Kirkuk, Iraq

## Abstract

In the title compound, C_21_H_24_N_4_O_2_, inversion-related mol­ecules are linked into dimers through pairs of N—H⋯O hydrogen bonds, which generate *R*_2_^2^(8) motifs. As well as dimer formation, an additional N—H⋯O hydrogen bond and two C—H⋯π contacts, involving H atoms from the phenyl ring and the pyrrole and benzene rings of the indole system, generate a three-dimensional network.

## Related literature

For the biological activity of indole acetic acid derivatives and indomethacin, see: Klassen (2001 ▶); Kirnura & Doi (1998 ▶); Rossiter *et al.* (2002 ▶); Shahab *et al.* (2009 ▶). For related structures, see: Trask *et al.* (2004 ▶); Gelbrich *et al.* (2007 ▶). For hydrogen-bond motifs, see: Bernstein *et al.* (1995 ▶).
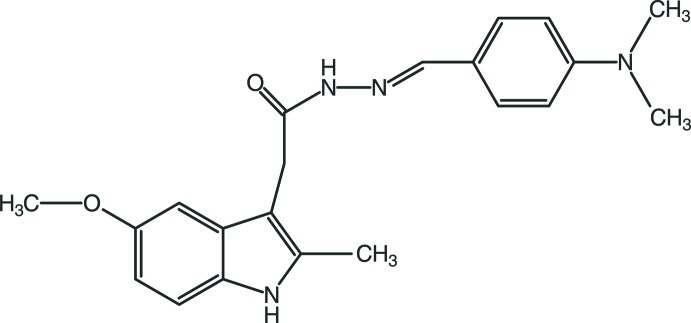


## Experimental

### 

#### Crystal data

C_21_H_24_N_4_O_2_
*M* *_r_* = 364.44
Monoclinic, 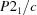

*a* = 9.600 (5) Å
*b* = 7.548 (4) Å
*c* = 25.802 (14) Å
β = 95.10 (1)°
*V* = 1862.2 (17) Å^3^
*Z* = 4
Mo *K*α radiation
μ = 0.09 mm^−1^
*T* = 100 K
0.10 × 0.01 × 0.01 mm

#### Data collection

Rigaku Saturn724+ diffractometer
Absorption correction: multi-scan (*CrystalClear*; Rigaku, 2001 ▶) *T*_min_ = 0.992, *T*_max_ = 0.999
10458 measured reflections
3280 independent reflections
2386 reflections with *I* > 2σ(*I*)
*R*_int_ = 0.048

#### Refinement

*R*[*F* ^2^ > 2σ(*F* ^2^)] = 0.069
*wR*(*F* ^2^) = 0.159
*S* = 1.15
3280 reflections
248 parameters
H-atom parameters constrained
Δρ_max_ = 0.21 e Å^−3^
Δρ_min_ = −0.27 e Å^−3^

### 

Data collection: *CrystalClear* (Rigaku, 2001 ▶); cell refinement: *CrystalClear* (Rigaku, 2001 ▶); data reduction: *CrystalClear*; program(s) used to solve structure: *SHELXS97* (Sheldrick, 2008 ▶); program(s) used to refine structure: *SHELXL97* (Sheldrick, 2008 ▶); molecular graphics: *ORTEP-3 for Windows* (Farrugia, 1997 ▶); software used to prepare material for publication: *WinGX* (Farrugia, 1999 ▶) and *PLATON* (Spek, 2009 ▶).

## Supplementary Material

Crystal structure: contains datablock(s) global, I. DOI: 10.1107/S1600536812026013/sj5239sup1.cif

Structure factors: contains datablock(s) I. DOI: 10.1107/S1600536812026013/sj5239Isup2.hkl

Supplementary material file. DOI: 10.1107/S1600536812026013/sj5239Isup3.cml

Additional supplementary materials:  crystallographic information; 3D view; checkCIF report

## Figures and Tables

**Table 1:** Hydrogen-bond geometry (Å, °) *Cg*1 and *Cg*2 are the centroids of the N1/C1–C3/C8 and C3–C8 rings, respectively.

*D*—H⋯*A*	*D*—H	H⋯*A*	*D*⋯*A*	*D*—H⋯*A*
N1—H1⋯O2^i^	0.88	2.21	2.984 (3)	147
N2—H2⋯O1^ii^	0.88	1.97	2.854 (3)	179
C18—H18⋯*Cg*2^iii^	0.95	2.84	3.692 (4)	151
C19—H19⋯*Cg*1^iii^	0.95	2.72	3.508 (4)	141

Symmetry codes: (i) 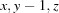; (ii) 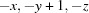; (iii) 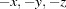.

**Fig. 1.**
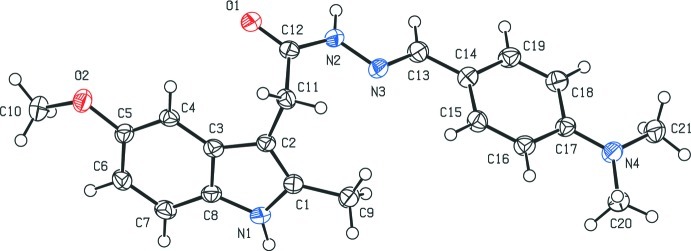
View of the title compound with the atom numbering scheme. Displacement ellipsoids for non-H atoms are drawn at the 50% probability level.

**Fig. 2.**
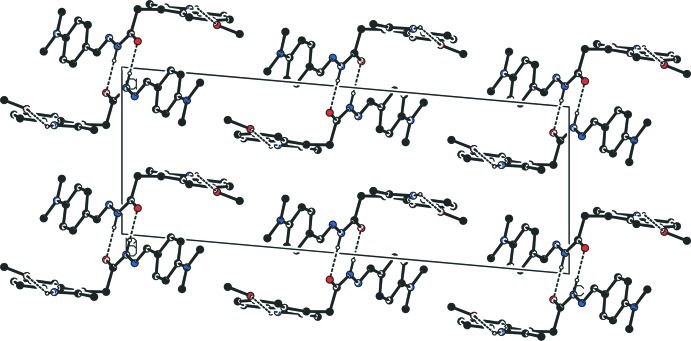
View of the molecular packing and the dimers formed through N—H···O hydrogen bonds of viewed along the b axis. Hydrogen atoms not involved in hydrogen bonding have been omitted for clarity.

## supplementary crystallographic information

### Comment

Indole-3-acetic acid (IAA) is the main auxin in plants, controlling many important physiological processes including cell enlargement and division, tissue differentiation, and responses to light and gravity (Shahab *et al*., 2009). In addition, derivatives of substituted indole-acetic acid are active oxidative pro-drugs with potential of cancer therapy (Rossiter *et al.*, 2002). Indomethacin is an example of IAA derivatives exhibits anti-inflammatory, analgesic, and antipyretic properties and is therefore used to treat acute and chronic pain (Klassen, 2001; Kirnura & Doi, 1998). As part of our interest in production of potential pharmaceutical active compounds based on well known pharmacophores e.g indomethacin, we are herein reporting the synthesis and crystal structure of the title compound.

In the title molecule (I), Fig. 1, the 1*H*-indole system (N1\C1—C8) is essentially planar [maximum deviation -0.025 (3) Å for atom C1] and makes a dihedral angle of 73.65 (12) ° with the (C14–C19) benzene ring. The bond lengths and angles are normal and comparable to those observed in the related structures (Trask *et al.*, 2004; Gelbrich *et al.*, 2007).

In the crystal structure, molecules form a dimer, in which a pair of N1—H1···O2 hydrogen bonds generate an intermolecular *R*_2_^2^(8) ring (Bernstein, *et al.*, 1995; Table 1, Fig. 2). These dimers are further linked by the N2—H2···O1 hydrogen bonds. Two additional C—H···π interactions also contribute to an extensive three dimensional network (Table 1).

### Experimental

A solution of 341 mg (1 mmol) 2-{1-[(4-chlorophenyl)carbonyl]-2-methyl-1*H*-indol-3-yl}acetohydrazide in 30 ml ethanol was added to a solution of 149 mg (1 mmol) 4-(dimethylamino)benzaldehyde in 20 ml ethanol in presence of few drops of catalytic glacial acetic acid and refluxed at 350 K for 6 h. On evaporating the excess solvent, a mass solid product was collected, washed with cold ethanol and dried. The crude product was recrystallized from ethanol to afford the title compound in a good yield (77%). Pure crystals suitable for X-ray diffraction were grown by slow evaporation of ethanol solution of the product at room temperature (m.p. 381 K).

### Refinement

All H-atoms were placed in calculated positions [N—H = 0.88 Å, C—H (aromatic) = 0.95 Å, C—H (methyl) = 0.98 Å and C—H (methylene) = 0.99 Å] and were refined by using a riding model approximation, with *U*_iso_(H) = 1.2 or 1.5 *U*_eq_(C,*N*).

### Crystal data

**Table:**

C_21_H_24_N_4_O_2_	*F*(000) = 776
*M**_r_* = 364.44	*D*_x_ = 1.300 Mg m^−^^3^
Monoclinic, *P*2_1_/*c*	Mo *K*α radiation, λ = 0.71075 Å
Hall symbol: -P 2ybc	Cell parameters from 5554 reflections
*a* = 9.600 (5) Å	θ = 2.5–31.2°
*b* = 7.548 (4) Å	µ = 0.09 mm^−^^1^
*c* = 25.802 (14) Å	*T* = 100 K
β = 95.10 (1)°	Needle, colourless
*V* = 1862.2 (17) Å^3^	0.10 × 0.01 × 0.01 mm
*Z* = 4	

### Data collection

**Table:**

Rigaku Saturn724+ diffractometer	3280 independent reflections
Radiation source: Rotating Anode	2386 reflections with *I* > 2σ(*I*)
Confocal monochromator	*R*_int_ = 0.048
Detector resolution: 28.5714 pixels mm^-1^	θ_max_ = 25.0°, θ_min_ = 3.1°
profile data from ω–scans	*h* = −11→10
Absorption correction: multi-scan (*CrystalClear*; Rigaku, 2001)	*k* = −8→8
*T*_min_ = 0.992, *T*_max_ = 0.999	*l* = −30→30
10458 measured reflections	

### Refinement

**Table:**

Refinement on *F*^2^	Primary atom site location: structure-invariant direct methods
Least-squares matrix: full	Secondary atom site location: difference Fourier map
*R*[*F*^2^ > 2σ(*F*^2^)] = 0.069	Hydrogen site location: inferred from neighbouring sites
*wR*(*F*^2^) = 0.159	H-atom parameters constrained
*S* = 1.15	*w* = 1/[σ^2^(*F*_o_^2^) + (0.0588*P*)^2^ + 0.6401*P*] where *P* = (*F*_o_^2^ + 2*F*_c_^2^)/3
3280 reflections	(Δ/σ)_max_ < 0.001
248 parameters	Δρ_max_ = 0.21 e Å^−^^3^
0 restraints	Δρ_min_ = −0.27 e Å^−^^3^

### Special details

**Table:**

Geometry. Bond distances, angles *etc*. have been calculated using the rounded fractional coordinates. All su's are estimated from the variances of the (full) variance-covariance matrix. The cell e.s.d.'s are taken into account in the estimation of distances, angles and torsion angles
Refinement. Refinement on *F*^2^ for ALL reflections except those flagged by the user for potential systematic errors. Weighted *R*-factors *wR* and all goodnesses of fit *S* are based on *F*^2^, conventional *R*-factors *R* are based on *F*, with *F* set to zero for negative *F*^2^. The observed criterion of *F*^2^ > σ(*F*^2^) is used only for calculating -*R*-factor-obs *etc*. and is not relevant to the choice of reflections for refinement. *R*-factors based on *F*^2^ are statistically about twice as large as those based on *F*, and *R*-factors based on ALL data will be even larger.

### Fractional atomic coordinates and isotropic or equivalent isotropic displacement parameters (Å^2^)

**Table:**

	*x*	*y*	*z*	*U*_iso_*/*U*_eq_	
O1	0.1627 (2)	0.5758 (2)	0.03536 (8)	0.0319 (7)	
O2	0.3022 (2)	0.7669 (2)	0.21269 (8)	0.0334 (7)	
N1	0.3847 (2)	0.0787 (3)	0.15126 (9)	0.0291 (8)	
N2	0.1218 (2)	0.3244 (3)	−0.00973 (9)	0.0294 (8)	
N3	0.1684 (2)	0.1639 (3)	−0.02855 (9)	0.0283 (8)	
N4	0.1656 (2)	−0.5960 (3)	−0.14532 (10)	0.0326 (9)	
C1	0.3807 (3)	0.1057 (4)	0.09794 (11)	0.0270 (9)	
C2	0.3661 (3)	0.2837 (3)	0.08759 (11)	0.0239 (8)	
C3	0.3558 (3)	0.3719 (4)	0.13661 (11)	0.0250 (9)	
C4	0.3339 (3)	0.5484 (4)	0.15053 (11)	0.0250 (9)	
C5	0.3263 (3)	0.5893 (4)	0.20232 (12)	0.0272 (9)	
C6	0.3425 (3)	0.4582 (4)	0.24098 (11)	0.0300 (10)	
C7	0.3625 (3)	0.2838 (4)	0.22774 (12)	0.0305 (10)	
C8	0.3676 (3)	0.2404 (4)	0.17560 (12)	0.0271 (9)	
C9	0.3920 (3)	−0.0469 (4)	0.06217 (12)	0.0319 (10)	
C10	0.2608 (3)	0.8097 (4)	0.26328 (12)	0.0358 (10)	
C11	0.3555 (3)	0.3737 (4)	0.03525 (11)	0.0274 (9)	
C12	0.2068 (3)	0.4328 (4)	0.01983 (11)	0.0262 (9)	
C13	0.0743 (3)	0.0759 (4)	−0.05616 (12)	0.0312 (10)	
C14	0.1025 (3)	−0.0925 (4)	−0.08031 (11)	0.0277 (9)	
C15	0.2347 (3)	−0.1724 (4)	−0.07641 (12)	0.0301 (10)	
C16	0.2558 (3)	−0.3363 (4)	−0.09783 (11)	0.0293 (10)	
C17	0.1459 (3)	−0.4293 (4)	−0.12538 (11)	0.0273 (9)	
C18	0.0138 (3)	−0.3465 (4)	−0.13131 (12)	0.0314 (10)	
C19	−0.0058 (3)	−0.1828 (4)	−0.10940 (12)	0.0316 (10)	
C20	0.2987 (3)	−0.6849 (4)	−0.13441 (12)	0.0331 (10)	
C21	0.0517 (3)	−0.6876 (4)	−0.17479 (13)	0.0372 (10)	
H1	0.39620	−0.02420	0.16710	0.0350*	
H2	0.03410	0.35570	−0.01740	0.0350*	
H4	0.32440	0.63850	0.12470	0.0300*	
H6	0.33980	0.48990	0.27650	0.0360*	
H7	0.37260	0.19470	0.25380	0.0370*	
H9A	0.30800	−0.12060	0.06220	0.0480*	
H9B	0.47450	−0.11770	0.07380	0.0480*	
H9C	0.40100	−0.00320	0.02690	0.0480*	
H10A	0.18490	0.73070	0.27160	0.0540*	
H10B	0.22840	0.93280	0.26350	0.0540*	
H10C	0.34080	0.79490	0.28930	0.0540*	
H11A	0.38590	0.29080	0.00870	0.0330*	
H11B	0.41840	0.47780	0.03660	0.0330*	
H13	−0.01760	0.12310	−0.06100	0.0370*	
H15	0.31160	−0.11210	−0.05860	0.0360*	
H16	0.34650	−0.38750	−0.09390	0.0350*	
H18	−0.06220	−0.40390	−0.15050	0.0380*	
H19	−0.09560	−0.12940	−0.11410	0.0380*	
H20A	0.37050	−0.62330	−0.15230	0.0500*	
H20B	0.29080	−0.80760	−0.14670	0.0500*	
H20C	0.32490	−0.68400	−0.09680	0.0500*	
H21A	−0.02660	−0.70150	−0.15320	0.0560*	
H21B	0.08360	−0.80470	−0.18520	0.0560*	
H21C	0.02110	−0.61870	−0.20590	0.0560*	

### Atomic displacement parameters (Å^2^)

**Table:**

	*U*^11^	*U*^22^	*U*^33^	*U*^12^	*U*^13^	*U*^23^
O1	0.0352 (12)	0.0256 (11)	0.0337 (12)	0.0026 (9)	−0.0034 (10)	−0.0007 (9)
O2	0.0435 (13)	0.0253 (11)	0.0317 (12)	−0.0023 (10)	0.0048 (10)	−0.0037 (9)
N1	0.0331 (14)	0.0228 (13)	0.0304 (15)	0.0052 (11)	−0.0033 (11)	0.0032 (11)
N2	0.0279 (13)	0.0251 (14)	0.0338 (15)	0.0055 (11)	−0.0052 (11)	−0.0033 (11)
N3	0.0326 (14)	0.0221 (13)	0.0301 (14)	0.0016 (11)	0.0026 (11)	0.0005 (11)
N4	0.0258 (13)	0.0269 (14)	0.0439 (17)	0.0008 (11)	−0.0031 (12)	−0.0045 (12)
C1	0.0215 (14)	0.0291 (16)	0.0300 (17)	0.0024 (13)	0.0003 (12)	−0.0029 (14)
C2	0.0187 (13)	0.0247 (15)	0.0277 (16)	0.0015 (12)	−0.0014 (12)	−0.0004 (13)
C3	0.0172 (13)	0.0268 (16)	0.0304 (17)	−0.0008 (12)	−0.0012 (12)	0.0006 (13)
C4	0.0257 (15)	0.0219 (15)	0.0267 (16)	−0.0021 (12)	−0.0021 (12)	0.0048 (13)
C5	0.0264 (15)	0.0220 (15)	0.0323 (17)	−0.0008 (13)	−0.0015 (13)	−0.0017 (13)
C6	0.0357 (17)	0.0310 (17)	0.0230 (16)	−0.0004 (14)	0.0018 (13)	0.0004 (14)
C7	0.0355 (17)	0.0294 (17)	0.0258 (17)	−0.0002 (14)	−0.0017 (13)	0.0062 (14)
C8	0.0240 (15)	0.0245 (15)	0.0317 (17)	0.0023 (13)	−0.0032 (12)	0.0015 (14)
C9	0.0319 (16)	0.0271 (16)	0.0362 (18)	0.0017 (13)	0.0011 (14)	−0.0028 (14)
C10	0.0412 (18)	0.0318 (17)	0.0348 (19)	−0.0010 (15)	0.0065 (15)	−0.0076 (15)
C11	0.0292 (15)	0.0269 (16)	0.0255 (16)	−0.0034 (13)	0.0000 (13)	0.0005 (13)
C12	0.0310 (16)	0.0252 (16)	0.0224 (16)	0.0015 (13)	0.0019 (13)	0.0040 (13)
C13	0.0286 (16)	0.0291 (17)	0.0355 (18)	−0.0004 (13)	0.0008 (14)	−0.0009 (14)
C14	0.0265 (15)	0.0263 (16)	0.0306 (17)	−0.0014 (13)	0.0047 (13)	0.0007 (13)
C15	0.0252 (15)	0.0313 (17)	0.0333 (18)	−0.0030 (13)	0.0001 (13)	−0.0028 (14)
C16	0.0239 (15)	0.0302 (17)	0.0337 (18)	0.0011 (13)	0.0016 (13)	0.0002 (14)
C17	0.0282 (15)	0.0235 (16)	0.0300 (17)	−0.0011 (13)	0.0018 (13)	0.0019 (13)
C18	0.0242 (15)	0.0295 (17)	0.0397 (19)	−0.0042 (13)	−0.0010 (13)	0.0015 (15)
C19	0.0247 (15)	0.0300 (17)	0.0396 (19)	0.0020 (13)	0.0009 (13)	0.0009 (14)
C20	0.0322 (17)	0.0320 (18)	0.0346 (19)	0.0042 (14)	0.0008 (14)	−0.0015 (14)
C21	0.0340 (17)	0.0291 (17)	0.047 (2)	−0.0033 (14)	−0.0047 (15)	−0.0053 (15)

### Geometric parameters (Å, º)

**Table:**

O1—C12	1.239 (3)	C15—C16	1.377 (4)
O2—C5	1.390 (3)	C16—C17	1.406 (4)
O2—C10	1.435 (4)	C17—C18	1.410 (4)
N1—C1	1.388 (4)	C18—C19	1.379 (4)
N1—C8	1.389 (4)	C4—H4	0.9500
N2—N3	1.394 (3)	C6—H6	0.9500
N2—C12	1.344 (4)	C7—H7	0.9500
N3—C13	1.285 (4)	C9—H9A	0.9800
N4—C17	1.379 (4)	C9—H9B	0.9800
N4—C20	1.449 (4)	C9—H9C	0.9800
N4—C21	1.451 (4)	C10—H10A	0.9800
N1—H1	0.8800	C10—H10B	0.9800
N2—H2	0.8800	C10—H10C	0.9800
C1—C2	1.375 (4)	C11—H11A	0.9900
C1—C9	1.486 (4)	C11—H11B	0.9900
C2—C3	1.441 (4)	C13—H13	0.9500
C2—C11	1.507 (4)	C15—H15	0.9500
C3—C4	1.401 (4)	C16—H16	0.9500
C3—C8	1.411 (4)	C18—H18	0.9500
C4—C5	1.380 (4)	C19—H19	0.9500
C5—C6	1.404 (4)	C20—H20A	0.9800
C6—C7	1.378 (4)	C20—H20B	0.9800
C7—C8	1.390 (4)	C20—H20C	0.9800
C11—C12	1.515 (4)	C21—H21A	0.9800
C13—C14	1.452 (4)	C21—H21B	0.9800
C14—C19	1.404 (4)	C21—H21C	0.9800
C14—C15	1.401 (4)		
			
C5—O2—C10	117.2 (2)	C5—C4—H4	121.00
C1—N1—C8	109.0 (2)	C5—C6—H6	120.00
N3—N2—C12	122.0 (2)	C7—C6—H6	120.00
N2—N3—C13	114.3 (2)	C6—C7—H7	121.00
C17—N4—C20	119.9 (2)	C8—C7—H7	121.00
C17—N4—C21	120.6 (2)	C1—C9—H9A	110.00
C20—N4—C21	119.5 (2)	C1—C9—H9B	110.00
C8—N1—H1	125.00	C1—C9—H9C	109.00
C1—N1—H1	126.00	H9A—C9—H9B	109.00
N3—N2—H2	119.00	H9A—C9—H9C	109.00
C12—N2—H2	119.00	H9B—C9—H9C	109.00
N1—C1—C2	109.2 (2)	O2—C10—H10A	109.00
N1—C1—C9	120.3 (3)	O2—C10—H10B	109.00
C2—C1—C9	130.5 (3)	O2—C10—H10C	110.00
C3—C2—C11	125.0 (2)	H10A—C10—H10B	109.00
C1—C2—C11	127.8 (3)	H10A—C10—H10C	109.00
C1—C2—C3	107.2 (2)	H10B—C10—H10C	109.00
C2—C3—C8	107.1 (2)	C2—C11—H11A	110.00
C4—C3—C8	119.4 (3)	C2—C11—H11B	110.00
C2—C3—C4	133.5 (3)	C12—C11—H11A	109.00
C3—C4—C5	118.9 (3)	C12—C11—H11B	109.00
O2—C5—C4	115.2 (3)	H11A—C11—H11B	108.00
O2—C5—C6	123.6 (3)	N3—C13—H13	119.00
C4—C5—C6	121.2 (3)	C14—C13—H13	119.00
C5—C6—C7	120.4 (3)	C14—C15—H15	119.00
C6—C7—C8	118.9 (3)	C16—C15—H15	119.00
N1—C8—C7	131.3 (3)	C15—C16—H16	119.00
C3—C8—C7	121.1 (3)	C17—C16—H16	119.00
N1—C8—C3	107.5 (3)	C17—C18—H18	120.00
C2—C11—C12	110.8 (2)	C19—C18—H18	120.00
N2—C12—C11	118.7 (3)	C14—C19—H19	119.00
O1—C12—N2	120.4 (3)	C18—C19—H19	119.00
O1—C12—C11	120.9 (3)	N4—C20—H20A	109.00
N3—C13—C14	122.8 (3)	N4—C20—H20B	109.00
C13—C14—C19	119.8 (3)	N4—C20—H20C	109.00
C15—C14—C19	116.9 (3)	H20A—C20—H20B	109.00
C13—C14—C15	123.3 (3)	H20A—C20—H20C	109.00
C14—C15—C16	121.5 (3)	H20B—C20—H20C	109.00
C15—C16—C17	121.4 (3)	N4—C21—H21A	109.00
N4—C17—C16	121.5 (3)	N4—C21—H21B	110.00
C16—C17—C18	117.4 (3)	N4—C21—H21C	109.00
N4—C17—C18	121.1 (3)	H21A—C21—H21B	109.00
C17—C18—C19	120.5 (3)	H21A—C21—H21C	109.00
C14—C19—C18	122.2 (3)	H21B—C21—H21C	109.00
C3—C4—H4	121.00		
			
C10—O2—C5—C6	−14.5 (4)	C4—C3—C8—C7	−2.2 (4)
C10—O2—C5—C4	165.7 (2)	C2—C3—C4—C5	178.5 (3)
C8—N1—C1—C2	2.1 (3)	C4—C3—C8—N1	178.4 (2)
C8—N1—C1—C9	−178.1 (2)	C2—C3—C8—C7	179.6 (3)
C1—N1—C8—C7	179.3 (3)	C3—C4—C5—C6	1.1 (4)
C1—N1—C8—C3	−1.3 (3)	C3—C4—C5—O2	−179.0 (2)
C12—N2—N3—C13	180.0 (3)	C4—C5—C6—C7	−1.9 (4)
N3—N2—C12—C11	−3.1 (4)	O2—C5—C6—C7	178.3 (3)
N3—N2—C12—O1	179.0 (2)	C5—C6—C7—C8	0.6 (4)
N2—N3—C13—C14	178.6 (3)	C6—C7—C8—C3	1.4 (4)
C20—N4—C17—C18	−174.1 (3)	C6—C7—C8—N1	−179.2 (3)
C21—N4—C17—C18	2.5 (4)	C2—C11—C12—O1	84.2 (3)
C21—N4—C17—C16	−178.4 (3)	C2—C11—C12—N2	−93.7 (3)
C20—N4—C17—C16	5.1 (4)	N3—C13—C14—C15	−1.1 (5)
C9—C1—C2—C3	178.2 (3)	N3—C13—C14—C19	179.1 (3)
N1—C1—C2—C11	−179.3 (3)	C13—C14—C15—C16	176.9 (3)
C9—C1—C2—C11	0.9 (5)	C19—C14—C15—C16	−3.3 (4)
N1—C1—C2—C3	−2.0 (3)	C13—C14—C19—C18	−177.2 (3)
C11—C2—C3—C8	178.5 (3)	C15—C14—C19—C18	3.0 (4)
C11—C2—C3—C4	0.7 (5)	C14—C15—C16—C17	1.1 (5)
C1—C2—C3—C4	−176.8 (3)	C15—C16—C17—N4	−177.7 (3)
C1—C2—C11—C12	105.6 (3)	C15—C16—C17—C18	1.5 (4)
C3—C2—C11—C12	−71.3 (4)	N4—C17—C18—C19	177.4 (3)
C1—C2—C3—C8	1.1 (3)	C16—C17—C18—C19	−1.8 (4)
C8—C3—C4—C5	0.9 (4)	C17—C18—C19—C14	−0.5 (5)
C2—C3—C8—N1	0.1 (3)		

### Hydrogen-bond geometry (Å, º)

Cg1 and Cg2 are the centroids of the N1/C1–C3/C8 and C3–C8 rings, respectively.

**Table:**

*D*—H···*A*	*D*—H	H···*A*	*D*···*A*	*D*—H···*A*
N1—H1···O2^i^	0.88	2.21	2.984 (3)	147
N2—H2···O1^ii^	0.88	1.97	2.854 (3)	179
C11—H11*A*···N3	0.99	2.42	2.814 (4)	103
C18—H18···*Cg*2^iii^	0.95	2.84	3.692 (4)	151
C19—H19···*Cg*1^iii^	0.95	2.72	3.508 (4)	141

Symmetry codes: (i) *x*, *y*−1, *z*; (ii) −*x*, −*y*+1, −*z*; (iii) −*x*, −*y*, −*z*.
